# Continuous neurophatic orofacial pain: A retrospective study of 23 cases

**DOI:** 10.4317/jced.52560

**Published:** 2016-04-01

**Authors:** Dídac Sotorra-Figuerola, Alba Sánchez-Torres, Eduard Valmaseda-Castellón, Cosme Gay-Escoda

**Affiliations:** 1 DDS. Student of Oral Surgery and Implantology Department. School of Dentistry, University of Barcelona. Barcelona, Spain; 2 DDS. Student of Oral Surgery and Implantology Department. School of Dentistry, University of Barcelona. Barcelona, Spain; 3 DDS, MS, PhD, EBOS. Professor of Oral Surgery. Professor of Master of Oral Surgery and Implantology, School of Dentistry, University of Barcelona; Researcher of the IDIBELL Institute. Barcelona, Spain; 4 MD, DDS, MS, PhD, EBOS. Chairman and Full Professor of Oral and Maxillofacial Surgery. Director of the Master of Oral Surgery and Implantology, School of Dentistry, University of Barcelona; Researcher/Coordinator of the IDIBELL Institute; Head of Oral and Maxillofacial Surgery Department of the Teknon Medical Center. Barcelona, Spain

## Abstract

**Background:**

To determine the clinical characteristics of Continuous Neuropathic Orofacial Pain in patients that suffer Persistent Idiopathic Facial Pain (PIFP), Painful Post-Traumatic Trigeminal Neuropathy (PPTTN) or Burning Mouth Syndrome (BMS) and to describe their treatment.

**Material and Methods:**

A retrospective observational study was made, reviewing the clinical history of the patients diagnosed with Continuous Neuropathic Orofacial Pain between 2004 and 2011 at the Orofacial Pain Unit of the Master of Oral Surgery and Implantology of the University of Barcelona and at the Orofacial Pain Unit of the Teknon Medical Center of Barcelona.

**Results:**

The average age of the patients with Continuous Neuropathic Orofacial Pain was 54.5, with a clear female predominance (86.9%, n=20). Of all patients, 60.9% (n=14) were suffering a PIFP, 21.7% (n=5) had a BMS and 17.4% (n=4) were presenting a PPTTN. The pain quality described by the patients with Continuous Neuropathic Orofacial Pain was oppressive (43.47%, n=10), widely represented by patients with PIFP, and burning (39.13%, n=9) being the only quality that described patients with BMS. The treatment carried out with the patients was only pharmacologic. The most used drugs for the treatment of PIFP and PPTTN were clonazepam (50%, n=9) and amitriptyline (44.44%, n=8). However, a 55.5% (n=10) of the patients with PIFP or PPTTN required the association of two or more drugs for a correct pain control. All the patients with BMS responded satisfactorily to clonazepam.

**Conclusions:**

Continuous Neuropathic Orofacial Pain is a little known condition among the general population, physicians and dentists. This favors a late diagnosis and inaccurate treatments which entail unnecessary suffering. It is important to inform both the general population and health professionals concerning this painful condition.

** Key words:**Continuous neuropathic orofacial pain, persistent idiopathic facial pain, painful post-traumatic trigeminal neuropathy, burning mouth syndrome, atypical odontalgia.

## Introduction

Neuropathic pain is one of the most frustrating conditions that challenge dental clinicians because a wrong diagnosis may involve the realization of incorrect treatments. According to the *International Association for the Study of Pain*, neuropathic pain is initiated or caused by a primary lesion or a nervous system dysfunction (1). Therefore, neuropathic pain represents a structural or functional anomaly in the peripheral or central nervous system while somatic pain is a damage alert system, predominantly (2).

There are two main types of orofacial neuropathic pain: episodic neuropathic pain (mostly represented by paroxysmal neuralgias) and continuous neuropathic pain. Continuous neuropathic pain differs from episodic neuropathic pain by presenting periods of high and low intensity without complete remission. The diagnostic key is the absence of a somatic source of pain (2,3). This descriptive retrospective study focuses on patients that suffer Persistent Idiopathic Facial Pain (PIFP), Painful Post-traumatic Trigeminal Neuropathy (PPTTN) or Burning Mouth Syndrome (BMS), all included in the group of Continuous Neuropathic Orofacial Pain.

The *International Classification of Headache Disorders of the International Headache Society* (IHS) includes PIFP, PPTTN and BMS in the group *Painful cranial neuropathies and other facial pains* (group 13) (4). Okeson *et al.* (2,3,5) divided Continuous Neuropathic Orofacial Pain in three types: centrally mediated pain, peripherally mediated pain and metabolite polyneuropathies. These authors, unlike IHS, classify PPTTN (formerly known as Anesthesia Dolorosa) as a peripherally mediated pain because they attribute it to nervous deafferentation and traumatic neuroma (2,3).

Persistent Idiopathic Facial Pain, previously called Atypical Facial Pain, is diagnosed by excluding all other pathologies that may cause facial pain in the affected area. PIFP is described as a persistent facial and/or oral pain, with varying presentations but recurring daily for more than 2 hours per day over more than 3 months, in the absence of clinical neurological deficit (4). It is a chronic form of facial pain that is normally continuous, deep and poorly located, of low to moderate intensity with sporadic episodes of intense pain (5,6). A dental cause has been excluded by appropriate investigations. If this pain is located in a teeth or near a teeth is named Atypical Odontalgia (2,4,7).

Painful post-traumatic trigeminal neuropathy is a deafferentation pain that results from a loss of normal afferent information that reaches the central nervous system (CNS). PPTTN is a traumatic neuralgia, generally due to a traumatic neuroma formation after a surgical procedure or trauma (2). The diagnostic criterion is unilateral facial or oral pain following trauma to the trigeminal nerve, with clinically evident positive (hyperalgesia, allodynia) and/or negative (hypoaesthesia, hypoalgesia) signs of trigeminal nerve dysfunction. Pain is located in the distribution of the same trigeminal nerve and it has to be developed within 3-6 months of the traumatic event (4).

Finally, Burning Mouth Syndrome is an intraoral burning or dysaesthetic sensation on the tongue, lips, gingiva and/or oral mucosa, recurring daily for more than 2 hours per day over more than 3 months, without clinically evident causative lesions. Oral mucosa is of normal appearance and clinical examination including sensory testing is normal (2,4,8,9).

The objective of this study is to determine the clinical characteristics of Continuous Neuropathic Orofacial Pain in patients that suffer PIFP, PPTTN or BMS and to describe their treatment.

## Material and Methods

A retrospective observational study was made by means of reviewing the clinical records of patients diagnosed with Continuous Neuropathic Orofacial Pain seen between 2004 and 2011 at the Orofacial Pain Unit of the Master of Oral Surgery and Implantology of University of Barcelona and the Orofacial Pain Unit of Teknon Medical Center of Barcelona. The study design was approved by the Research and Ethics Committees of University.

The patients included were under clinical diagnosis of PIFP, PPTTN and BMS, according to group 13 of the *International Classification of Headache Disorders of the International Headache Society*. The patients with incomplete protocols were excluded.

The variables collected were divided in three categories:

- Patient variables: gender, age at the moment of diagnosis and the presence of systemic disease or psychological/psychiatric disorders.

- Pain variables: clinical diagnosis, quality, intensity, anatomical location, duration, pain aggravators and relievers, the possible trigger, time of evolution to clinical diagnosis and the concomitant symptoms of pain.

- Therapeutic variables: prescribed drug treatment and its side effects.

The patient data and the pain characteristics that were used for the statistical study were registered during the first visit to the Orofacial Pain Units. All the patients were subjected to the same protocolized clinical history.

The quality of pain was registered by showing the patients a list of descriptive adjectives: oppressive, electrical, burning, shooting, sharp and throbbing. In order to register the pain intensity, the patients were asked to classify it as low, moderate or intense.

 Table 1,  Table 2 and  Table 3 show the pain characteristics of each patient according to the clinical diagnosis: PIFP, PPTTN or BMS.

**Table 1:**
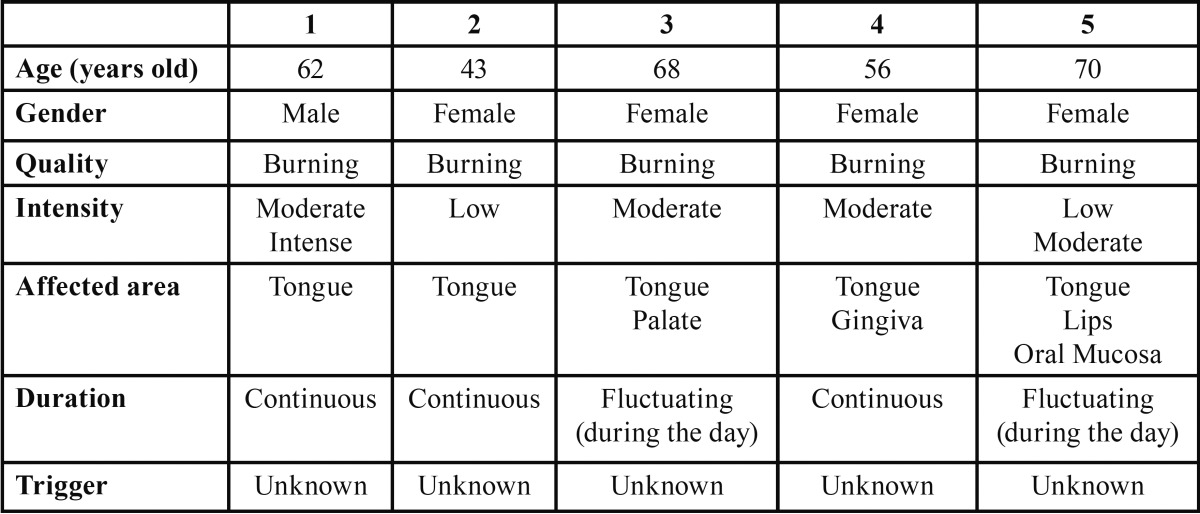
Characteristics of pain in patients with Burning Mouth Syndrome.

**Table 2:**
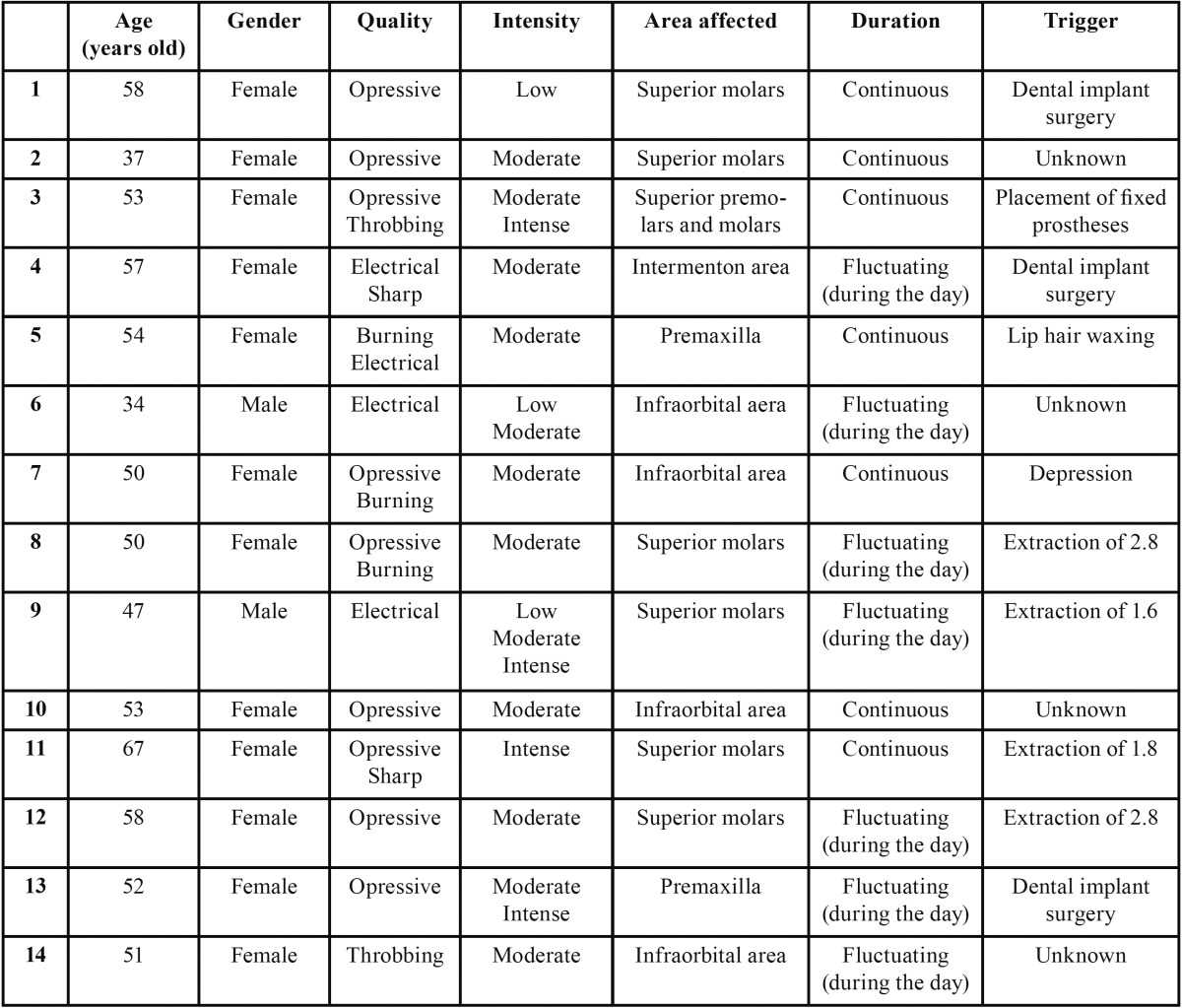
Characteristics of pain in patients with Persistent Idiophatic Facial Pain.

**Table 3:**
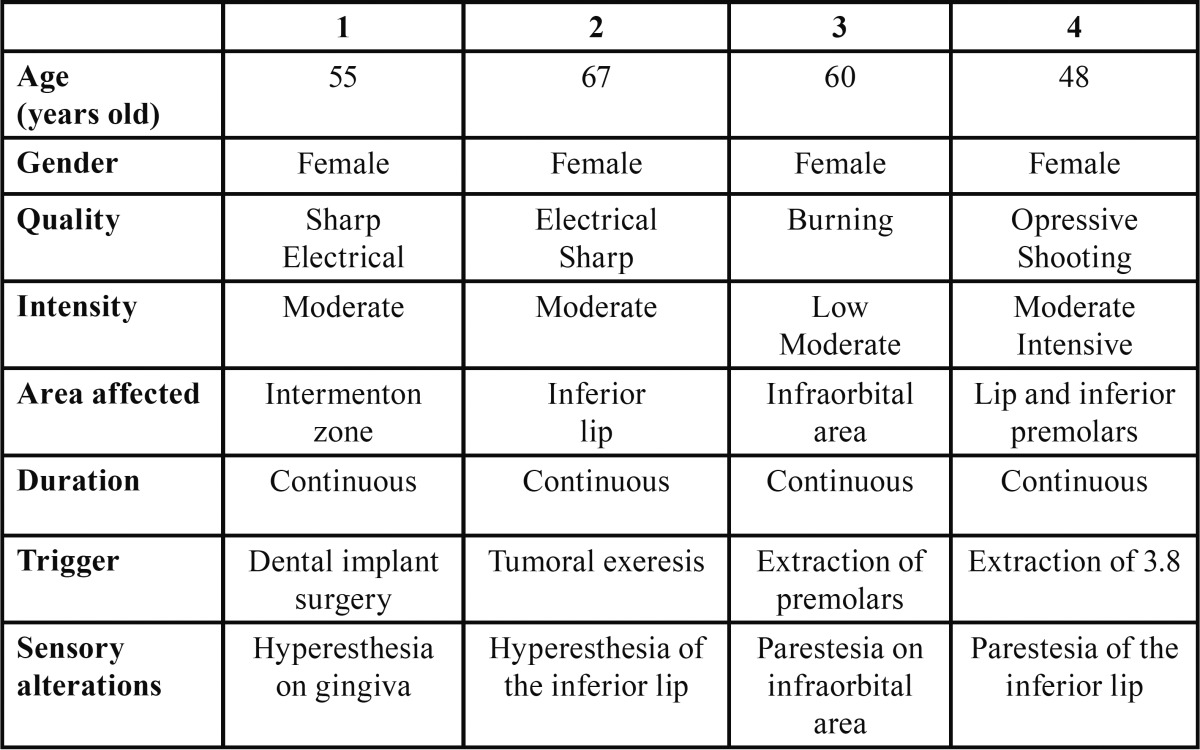
Characteristics of pain in patients with Anesthesia Dolorosa.

Microsoft Access for Windows was used for data collection. A descriptive analysis was performed with the Statistical Package for the Social Sciences for Windows (SPSS v15.0; SPSS Inc. Chicago, IL, USA, University of Barcelona license).

## Results

The study sample consisted of 23 patients with Continuous Neuropathic Orofacial Pain, with a clear female predominance (86.9%, n=20). The average age of patients was 54.5 (range 34-70). Of all patients, 60.9% (n=14) were suffering a PIFP, 21.7% (n=5) had a BMS and 17.4% (n=4) were presenting a PPTTN. The mean evolution time, namely the period between onsets of the symptoms and diagnosis, was 28.1 months (ranging from 0.5 to 96).

PIFP were located at the maxilla in 64.3% (n=9) of cases, at the infraorbital area in 28.6% (n=4) and one case (7.1%, n=1) at the intermenton (chin) zone. The location of PPTTN was 50% (n=2) on the inferior lip, and 25% (n=1) on the intermenton zone and the infraorbital zone. All the BMS were located in the tongue (100%, n=5), although three patients also referred having burning pain in the palate, oral mucosa and lips.

The pain quality described by the patients with Continuous Neuropathic Orofacial Pain was (n=23): oppressive (43.47%, n=10), widely represented by patients with PIFP, burning (39.13%, n=9), being the only quality that described patients with BMS, electrical (26.08%, n=6), sharp (17.39%, n=4), throbbing (8.69%, n=2) and 1 patient with PPTTN referred shooting pain (4.34%, n=1). The 39.13% (n=9) of cases reported two or more types of pain quality.

The most prevalent pain intensity was moderate (86.9%, n=20), followed by low (26.08%, n=6) and severe intensity (26.08%, n=6). Variations of pain intensity were described by 34.78% (n=8) of participants.

Stress increased the pain intensity for 34.8% (n=8) of cases and 43.5% (n= 10) of the patients presented other systemic diseases and psychiatric/psychological disorders related or not to the chronic pain.

All the patients with PPTTN (n=4) reported that their pain was triggered by a surgical procedure on the affected nervous zone and all the patients presented sensory alterations on the affected area (hyperesthesia, dysesthesia or paresthesia). The 71.43% (n=10) of patients that suffered a PIFP reported a pain trigger, for instance a surgical procedure, depression or lip hair waxing. No patients with PIFP presented sensibility alterations on the painful area. The patients with BMS did not report any pain triggering.

The treatment carried out with the patients was only pharmacologic. We divided the patients into two therapeutic groups. The first group describes the treatments performed for patients with PIFP and PPTTN. Patients with BMS comprised the second group.

PIFP and PPTTN (n=18) patients may often respond similarly to the same drug treatment, interindividual differences being essential. Association of two or more drugs for the correct pain control was required for 55.5% (n=10) of these patients. The 50% (n=9) of cases were treated with clonazepam, 44.44% (n=8) received amitriptyline and 33.33% (n=6) duloxetine. Besides, venlafaxine (n=1, 5.55%), nortriptyline (n=1, 5.55%), pregabalin (n=1, 5.55%) and gabapentine (n=1, 5.55%) were used. The drug most associated with the other ones was clonazepam. The drug of choice that we first used in all the patients of this group was amitriptyline. The most described side effects were drowsiness (n=2), fatigue (n=3), xerostomia (n=4), cardiovascular (n=2) and sensory alterations (n=2), among others.

All the patients with BMS (n=5) responded satisfactorily to treatment with clonazepam, at doses of 1-1.5 mg/day. The described side effects were somnolence (n=2), fatigue (n=1) and aggressiveness (n=1).

## Discussion

To date, the prevalence of PIFP in the general population is still unknown. According to this study, a higher prevalence of PIFP has been found among women. The highest prevalence of cases reported by literature is around the fourth decade of life, unlike in our sample that appears in the fifties (3,7,10). Although all ages may be affected by this painful condition, a case of impairment in childhood has not yet been described in literature. In our study, the youngest patient was 34 years old (7). Atypical Odontalgia presents as tooth pain or pain in a site where a tooth was extracted, in the absence of clinical and radiographic evidence of tooth pathology (11-13). PIFP has been described to occur in 3% to 6% of patients who undergo endodontic treatment (11,14). However, any patients in our study referred a previous endodontic treatment as a pain trigger. The referred causes were an oral surgery procedure (tooth extraction, periapical surgery and dental implant surgery) and other non-dental related causes such as history of depression, sinusitis and lip hair waxing. Although PIFP has been very little related to dental implant placement in the whole literature, we present four cases (15).

According to this work, some studies report that molars and premolars are the most frequently involved teeth and that the maxilla is more often affected than the mandible (7,16,17).

Regarding to PPTTN, it has not been described to have a gender tendency. All the patients have sensory alterations (hyperesthesia, dysesthesia or paresthesia) and relate that the start of pain was a surgical procedure like dental implant surgery (3,4,18). Renton and Yilmaz (12) reported a sample of 90 patients having iatrogenic lesions of inferior alveolar nerve: 60% appeared after third molar extraction, 19% after inferior alveolar block anaesthesia, 18% after dental implant placement and 8% were associated with endodontic treatment. These patients described allodynia, paresthesia, dysesthesia and hyperesthesia (2-4).

According to the available literature, tricyclic antidepressants (TCAs) seem to be the most effective medication for the treatment of neuropathic chronic orofacial pain (7,19-21). TCAs such as amitriptyline inhibit the recapture of serotonin and norepinephrine, neurotransmitters which are known to be present in CNS sites involved in pain inhibition (22). Therefore, it is suggested that TCAs could mediate therapeutic effects by increasing the activity of CNS pain inhibitory mechanism. Additionally, amitriptyline has affinity for muscarinic, histaminic and β-adrenergic receptors (14). Maybe this lower affinity is the responsible of its therapeutic effect. The amitriptyline, unlike other TCAs used in the treatment of chronic neuropathic pain (such as nortriptyline and desipramine), presents a high affinity for the above mentioned receptors (23). Other studies show that β-blockers and some anti-convulsants as clonazepam, gabapentine and baclofen seem to be fairly effective in the treatment of continuous neuropathic orofacial pain (15,19,24). Topical medications such as capsaicin, at a concentration of 0.025%, also give good results in certain patients with this disorder (2,15).

In agreement with these findings, the most used drugs in our patients diagnosed with PPTTN and PFIP were clonazepam and amitriptyline. The drug of choice that we first used in all these patients was amitriptyline. However, in a third of patients with PPTTN and PIFP, we had to prescribe two or more drugs to achieve an adequate pain control.

**Figure 1:**
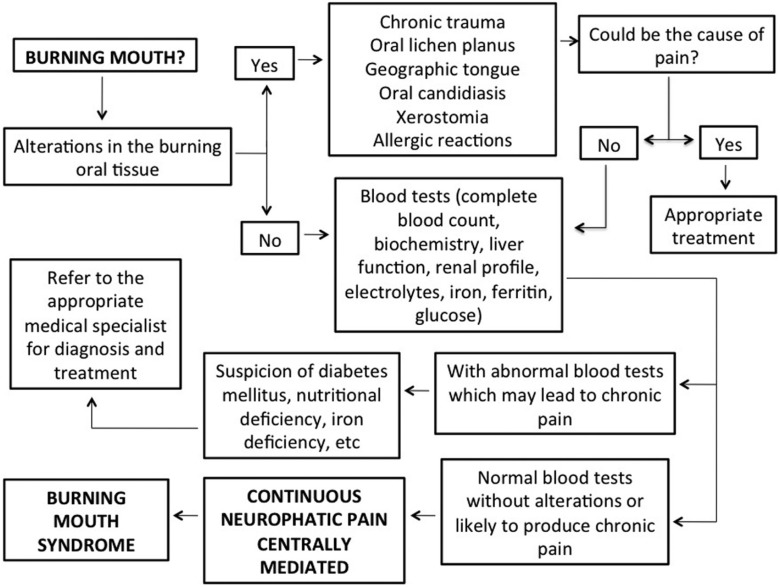
Proposal of criteria to consider in establishing the diagnosis of Burning Mouth Syndrome.

BMS is a painful intraoral sensation characterized by the burning sensation of the tongue, lips, gingiva and/or oral mucosa (2). All the patients of our study were females (n=5) and described a burning pain on the tongue; besides, some of them referred pain on the lips, gingiva and palate. The literature shows that the age of presentation of BMS varies from 50 to 70 years old, being rare before the third decade (2,25). Typically, it initiates around or after the menopausal period. Our group had an average age of 59.8 years old; the youngest patient was 43 years old. The intensity of pain ranges from low to intense, appearing while waking up or later during the day. BMS does not cause incapacity and it usually has a sudden onset (2,8). The appearance of the oral soft tissue is normal or reveals few clinical abnormalities (2). From our patients, only one had an oral lichen planus on the painful area. The diagnostic criteria that we used are described in figure 1.

Clonazepam is the most commonly reported drug in literature to treat BMS (2,26,27). This anticonvulsant benzodiazepine is often used with an initial dose of 0.25 mg/day and after, the dose increases to 0.25 mg/week to achieve adequate pain control, with a maximum of 3 mg/day (2,26). Heckmann *et al.* (27) performed a randomized placebo-controlled clinical trial on clonazepam for the treatment of BMS and proved its therapeutic efficacy. Other drugs, such as TCAs (excluding amitriptyline because it induces hyposialia), gabapentine, paroxetine and chlordiazepoxide have been proposed for the treatment of BMS but with lower efficacy than clonazepam (27-29). In our study, pain was correctly controlled in all patients with BMS by means of clonazepam.

A recent study by Yang and Huang (30) proposes the use of a soft laser for the control of pain in BMS. They recommend the application of 1 to 7 sessions of laser. They describe a decreasing in pain of 47.6%. A similar result was reached in another study on the use of soft lasers in the treatment of pain in BMS (31).

## Conclusions

Continuous neuropathic orofacial pain is a little known condition among the general population, physicians and dentists. Unfortunately, this favors a late diagnosis and an inaccurate treatment which entails unnecessary suffering for the patient. It is important to inform the general population and health professionals concerning this painful condition.
